# Electrode Structuring via Carbon Nanotubes and Nafion Ionomer–Coated TiO_2_ Enhances the Durability of Proton Exchange Membrane Fuel Cells Under Carbon Corrosion Conditions

**DOI:** 10.1002/smll.202409650

**Published:** 2025-03-25

**Authors:** Ohsub Kim, Katie Heeyum Lim, JunHwa Kwon, Sung Jong Yoo, Jin Young Kim, Sung Ki Cho, Hyun S. Park, So Young Lee, Bora Seo, Myeong‐Geun Kim, Jong Hyun Jang, Hee‐Young Park

**Affiliations:** ^1^ Center for Hydrogen and Fuel Cells Korea Institute of Science and Technology (KIST) Seoul 02792 Republic of Korea; ^2^ Division of Energy & Environment Technology KIST School University of Science and Technology (UST) Seoul 02792 Republic of Korea; ^3^ Green School Korea University Seoul 02841 Republic of Korea

**Keywords:** carbon corrosion, mass transport, micrometer‐scale pore, proton exchange membrane fuel cell (PEMFC), structural additive

## Abstract

The widespread implementation of proton exchange membrane fuel cells (PEMFCs) is being delayed by their inadequate durability, particularly that of the cathode. To address this problem, two corrosion‐resistant additives are introduced to mitigate the structural collapse of the PEMFC cathode due to carbon corrosion: carbon nanotubes (CNTs) and a composite consisting of TiO_2_ coated with the Nafion ionomer (NT composite). CNTs give rise to a micrometer‐scale porous structure and improve the durability of the electrodes by preserving their structure during PEMFC operation. In tandem with the CNTs, the NT composite reinforces the structure with sub‐micrometer‐scale pores and maintains a stable ionomer distribution to promote gas transport and proton transfer within the electrode, respectively. The textured and reinforced structure is maintained even after carbon corrosion, thus significantly increasing the durability of PEMFCs, with a performance degradation of only 0.3% (i.e., the durability is ≈37 times higher) after accelerated durability tests. Moreover, the initial performance is comparable to that of state‐of‐the‐art electrodes with the conventional Pt/C catalyst. The significant enhancement of the durability of PEMFCs by creating an advanced electrode structure with suitable additives is expected to facilitate their development for various applications and practical commercialization.

## Graphical abstract

Carbon nanotubes and Nafion‐coated TiO_2_ composites, as structural additives for the cathode, enhance PEMFC durability under carbon corrosion conditions. They form micrometer‐scale pores after accelerated tests to improve mass transport. The electrode demonstrates a 37 times improvement in durability, offering a cost‐effective solution to extend the PEMFC lifespan without new catalyst synthesis, facilitating practical commercialization.

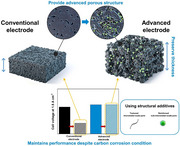

## 1 Introduction

The increasing demand to replace polluting combustion engines for vehicular transport has focused attention on alternative cleaner forms of power. In particular, proton exchange membrane fuel cells (PEMFCs) are considered promising next‐generation devices to generate clean energy.^[^ ^1^, ^2^ ^]^ Owing to their environmental friendliness, high power density, and high efficiency, PEMFCs have been extensively investigated for transportation applications; vehicles equipped with PEMFCs have been introduced to the market by several car manufacturers.^[^ ^3^ ^]^ Unfortunately, PEMFC‐equipped vehicles are not yet widely commercialized because the challenges associated with the cost and durability of PEMFC components have not been adequately addressed.^[^ ^4^, ^5^ ^]^

The durability of PEMFCs is critical because it not only determines the lifetime of the system but also has a significant impact on the cost of large‐scale commercialization. In particular, highly durable components can decrease the cost of maintenance and reduce the burden on consumers, thus lowering the barrier to purchase.^[^ ^6^ ^]^ The roadmap proposed by the U.S. Department of Energy (DOE) provides various strategies to overcome the barriers presented by the low durability and cost, such as the “activity loss due to the corrosion of the catalyst support.”^[^ ^6^ ^]^ Among the seven barriers listed therein, four are related to the durability of the electrode. Despite the considerable impact of electrode durability, sufficient improvements have not been realized thus far.

The electrochemical corrosion of carbon supports has a severe impact on electrode durability by causing high oxygen transfer resistance due to structural degradation.^[^ ^7^, ^8^, ^9^ ^]^ As the carbon support is electrochemically oxidized to CO_2_, the porous structure of the catalyst layer cannot be sustained and consequently collapses, thereby impeding mass transport.^[^ ^10^ ^]^ In particular, the ability to sustain mass transport, which determines the cell performance under high‐current conditions, is adversely affected because the supply of reactants is disrupted by water (a product of the electrochemical reaction) and the collapsed electrode structure.^[^ ^11^, ^12^, ^13^ ^]^ For example, the DOE Fuel Cell Consortium for Performance and Durability performed a carbon support accelerated stress test with an actual membrane electrode assembly (MEA) from Toyota Mirai fuel‐cell electric vehicles. They demonstrated that the cathode thickness decreased by 70% by the end of the test, which simultaneously lowered the overall performance (by approximately one‐tenth).^[^ ^14^ ^]^ Moreover, the five participating U.S. national laboratories confirmed the result that structural degradation is the main mechanism by which carbon corrosion increases oxygen transport resistance.^[^ ^8^ ^]^ These findings clearly indicate that the structural collapse needs to be mitigated to improve the durability of the PEMFC cathode against carbon corrosion.

Two major strategies have emerged to improve the durability of the electrode structure. The most recent studies have mainly focused on the development of new support materials to replace the conventional Pt/C to eliminate the factor responsible for the actual electrochemical degradation (i.e., carbon corrosion), rather than concentrating on structural stability. However, despite efforts to improve the durability by preventing the electrode from undergoing carbon corrosion, the initial performance could only be improved to a limited extent. For example, carbon‐based materials (e.g., graphitic carbon, CNTs, and carbon nanofibers (CNFs))^[^ ^15^, ^16^, ^17^, ^18^, ^19^, ^20^, ^21^, ^22^, ^23^, ^24^, ^25^ ^]^ and non‐carbon‐based supports (e.g., TiO_2_, SnO_2_, ITO, SiO_2_, WO_3_, TiN, TiC, and BN)^[^ ^26^, ^27^, ^28^, ^29^, ^30^, ^31^, ^32^, ^33^, ^34^, ^35^, ^36^, ^37^, ^38^, ^39^, ^40^ ^]^ were introduced and analyzed, typically by conducting half‐cell tests. Among these studies, several materials, specifically Pt/GC,^[^ ^15^ ^]^ Pt/CNT,^[^ ^16^, ^17^ ^]^ Pt/CNF,^[^ ^21^ ^]^ and Pt/TiO_2_,^[^ ^36^ ^]^ have enabled improved durability (2–17 times higher than that of Pt/C using their own system as reference). However, these materials had limited initial performance: the current density was 10%–16% lower than that with the conventional Pt/C catalyst at 0.6 V. These results demonstrate the limitations of the strategy focusing on materials development to improve the durability under carbon corrosion conditions while maintaining high initial performance because of the trade‐off that exists between the two. They also highlight the need for studies that focus on improving the durability of the conventional Pt/C electrode.

The second strategy involves the development of new approaches that focus on advanced Pt/C electrode structures to improve the durability of PEMFCs. In a few studies, ways to advance the electrode structure were investigated by forming textured micrometer‐scale pores as cracks or grooves in the electrode (textured electrode), which have been shown to improve mass transport.^[^ ^41^, ^42^, ^43^ ^]^ Despite the use of conventional Pt/C as the active electrode material, the durability yielded by this approach was 1.4–1.7 times higher because the textured structure of the electrode was maintained after carbon corrosion, and the initial current density was 4%–24% higher at 0.6 V. These results suggest that structural improvement is a valuable strategy to improve both the durability and initial performance of PEMFCs. Other research groups enhanced the Pt/C electrode by introducing structural additives that form reinforced sub‐micrometer‐scale pores (reinforced electrodes). This approach improved the mass transport and mitigated the structural deformation, as confirmed by the durability tests; the durability was 1.2–3 times higher because of the reinforced structure that remained after carbon corrosion.^[^ ^44^, ^45^ ^]^ However, the approach followed by Jung et al.^[^ ^44^ ^]^ entailed a trade‐off between performance and durability (the initial current density was 15% lower than that of the conventional Pt/C electrode (CE)), whereas their other approach increased the initial current density by 7% at 0.6 V.^[^ ^44^ ^]^ More specifically, Jung et al. fabricated a reinforced electrode with additional ionomer content as a structural additive to improve the structural stability after carbon corrosion. The durability of their electrode was 1.6 times higher in terms of the electrode thickness after carbon corrosion; however, the initial current density was lower than that of the conventional Pt/C electrode.^[^ ^44^ ^]^ In our previous study, we introduced a Nafion ionomer‐coated TiO_2_ composite (NT composite) as an additive material both to mitigate structural degradation by forming sub‐micrometer‐scale pores that were maintained even after the accelerated durability test (ADT), and to provide an additional ion pathway. The potential of the reinforced structural design of this electrode was demonstrated by its high durability after carbon corrosion.^[^ ^45^ ^]^ However, the limitations of the textured electrode include areas that can be exposed to structural collapse, which can lead to unnecessary degradation in the current density of 9%–20%. To counteract this limitation, we attempted to further improve the durability by combining a textured electrode with the concept of a reinforced electrode. This would take into consideration that reinforced electrodes are three times more durable because of their ability to mitigate structural degradation after carbon corrosion.

The need to increase both performance and durability by improving mass transport and mitigating structural collapse led us to propose the incorporation of design elements with both a textured structure (micrometer‐scale pores) and a reinforced structure (sub‐micrometer‐scale pores). Interestingly, CNTs were previously introduced as suitable additives for structural reinforcement by forming micrometer‐scale pores to improve mass transport, which can ultimately improve the initial performance.^[^ ^46^, ^47^, ^48^ ^]^ Although CNT reinforcement was observed to benefit only the initial performance, we expected it to be possible to use this additive in combination with the NT composite to improve the durability of PEMFCs by improving the mass transport after carbon corrosion while retaining both the micrometer‐scale and sub‐micrometer‐scale pores.

Herein, we propose a novel and practical concept for an advanced electrode consisting of conventional Pt/C and structural additives, designed to improve both the initial performance and PEMFC durability by preventing structural collapse and improving mass transport. The combined textured and reinforced structure (consisting of both micrometer‐scale and sub‐micrometer‐scale pores) of the Pt/C electrode was generated with the aid of CNT and NT composite additives. Optimization of the CNT ratio enabled us to control the pore formation in the electrode. The effects of the additives were analyzed by conducting various electrochemical analyses and using physical analysis techniques. This concept of advanced electrode design involving the use of structural additives (CNTs and NT composites) in conjunction with conventional Pt/C is expected to improve the lifetime of PEMFCs by ensuring high initial performance and support the commercialization of PEMFCs, as additional processes to manufacture or synthesize new catalysts are unnecessary. This strategy is a promising approach to address both the performance and durability challenges associated with PEMFCs.

## 2 Results and Discussion

**Figure 1:**
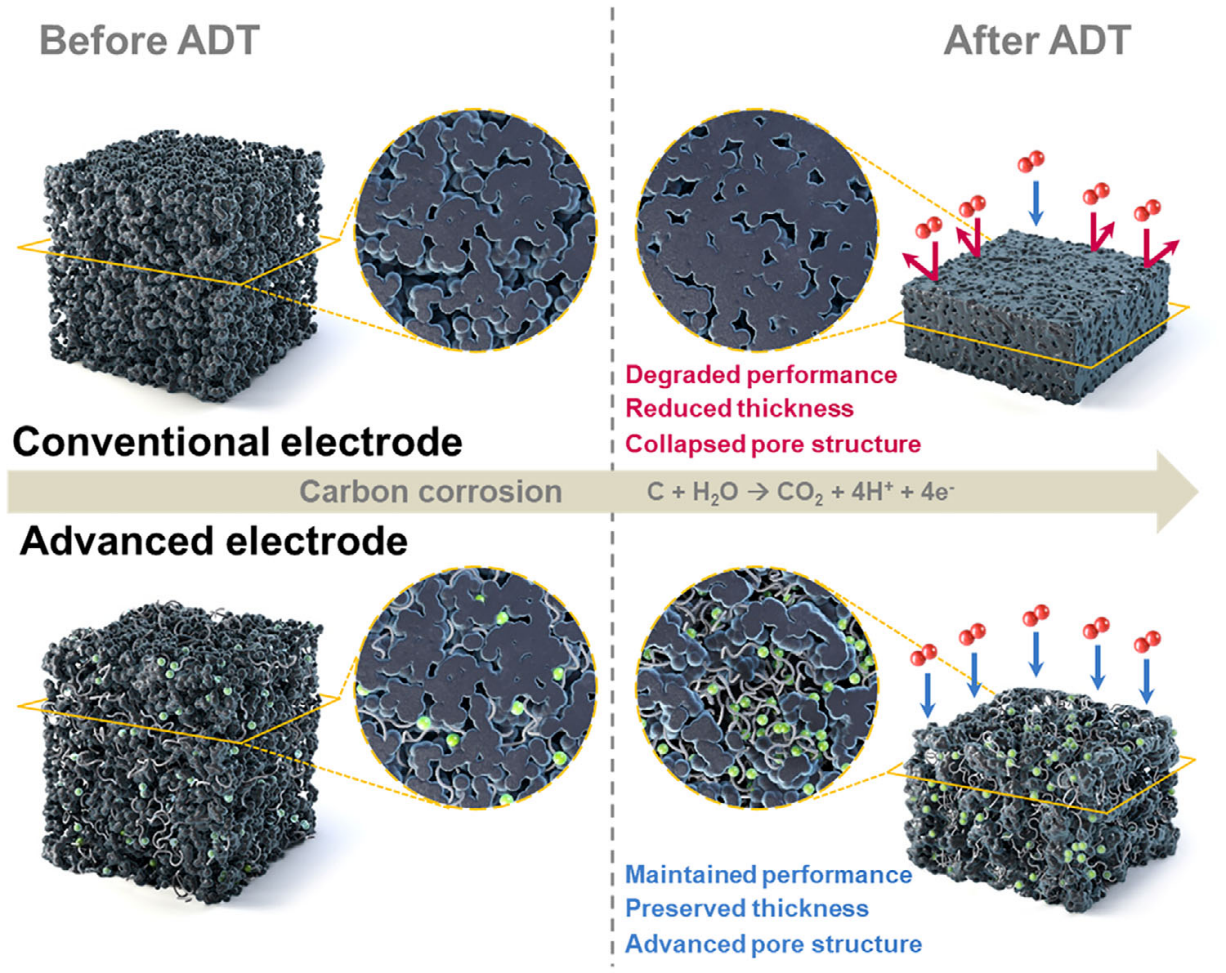
Schematic of the conventional and structurally reinforced (CN2) PEMFC cathodes before and after the accelerated durability test (ADT). The structurally reinforced cathode incorporates CNTs and TiO_2_ coated with the Nafion ionomer.

The concept of a reinforced electrode that incorporates CNTs and NT composite as structural additives is illustrated in **Figure** **1**. After the ADT, the reinforced electrode not only forms a structure with micrometer‐scale pores, which improves oxygen transport through the structure; its thickness is also preserved, such that structural degradation is reduced. In contrast, the CE undergoes severe structural deformation during PEMFC operation, which substantially degrades the performance of the PEMFC. The composition of the advanced electrode that incorporates the CNTs and NT composite was formulated based on the hypothesis that these structural additives not only deliver high initial performance in conjunction with conventional Pt/C, but also form a micrometer‐scale porous electrode structure with CNTs after carbon corrosion, thereby improving the oxygen transport to the oxygen reduction reaction. In addition, the NT composite is expected to provide a stable pathway for facile proton transfer through the entire catalyst layer, as demonstrated in our previous study.^[^ ^45^ ^]^ The Pt/C‐based catalysts are labeled based on the volumetric ratio of the CNTs: CN1 (0.5 vol.%), CN2 (1.25 vol.%), CN3 (2.5 vol.%), and CN4 (5.0 vol.%)).

**Figure 2:**
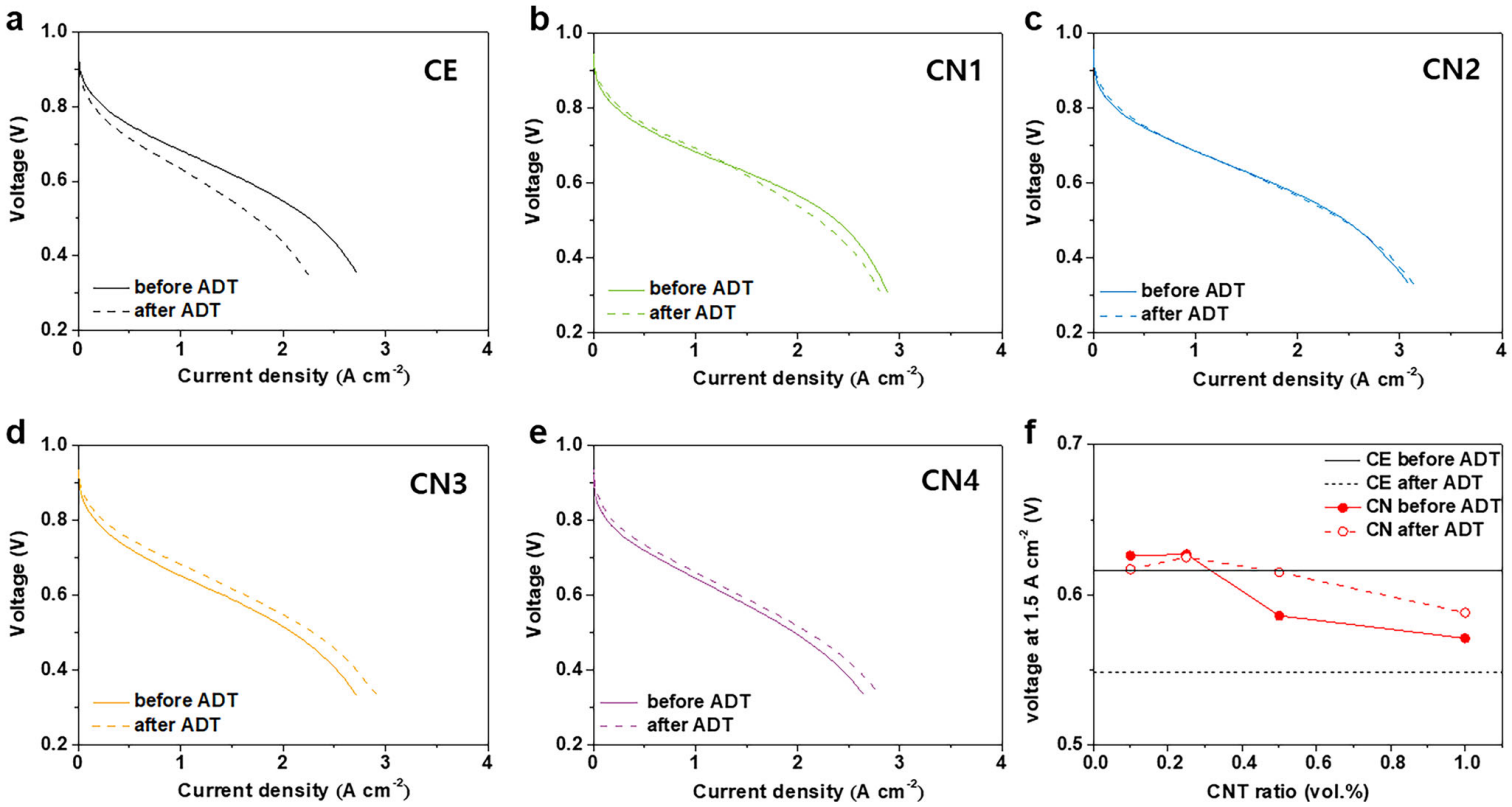
Polarization curves of MEAs with a) CE, b) CN1, c) CN2, d) CN3, and e) CN4; f) comparison of the cell voltage (at 1.5 A cm^−2^) for the respective MEAs before and after ADT.

**Figure** **2** presents the polarization curves and PEMFC performance (at a current density of 1.5 A cm^−2^) of each electrode before and after the ADT. The performance of CN2 is the highest after the ADT among the CN samples, which may be the result of the optimization of the additive ratio for the structurally reinforced electrode. For reference, the initial single‐cell performance of CE is 0.62 V at 1.5 A cm^−2^ (Figure 2a). In contrast, the initial performance of CN2 (with a CNT ratio of 1.25 vol.%) is 0.63 V at 1.5 A cm^−2^ (Figure 2c). In addition, CN1 and CN2 outperform CE in the high current density region, thereby demonstrating the positive effect of structural additives on mass transport. However, in the presence of an excess amount of CNT, as in CN4, the performance decreases markedly to 0.57 V (Figure 2e), which is lower than that of the CE. Thus, an optimal ratio of additives is required to improve the initial performance of the electrode. Moreover, the performance degradation after the ADT appears to be considerably mitigated by the addition of the CNTs and NT composite, particularly at the high current density that is dominant during mass transport. Figure 2f compares the overall trend of the performance of CNs both before and after the ADT, with the CE line based on 1.5 A cm^−2^. At the end of the ADT, the voltage levels produced by all the CN samples remain higher than that of the CE. Compared with the CE, which exhibits a loss of 68 mV (−11% compared with the voltage before the ADT), the voltage losses experienced by CN1 and CN2 are only 9 mV (−1.4%) and 2 mV (−0.3%) at 1.5 A cm^−2^ after the ADT, respectively. Although CN3 and CN4 demonstrate limited initial performance, their performance has improved by the end of the ADT. Moreover, all the CNs experience less degradation in the high current density region, during which the structures of the electrodes maintain their porosity for mass transport even after carbon corrosion. Thus, the CNT and NT composites successfully serve as structurally reinforcing additives to improve the mass transport both before and after carbon corrosion.

Furthermore, among the CN samples, CN2 exhibits the lowest performance degradation after the ADT.

To demonstrate the effect of the CNT ratio while maintaining a constant amount of NT composite, Figure S9 (Supporting Information) showed 0.62 V at 1.5 A cm^−2^ which is 30 mV (−4.5%) performance loss after ADT. This comparison indicates that CN2 can reduce the single‐cell performance degradation rate after ADT by 97%, with 59% of the effect attributable to the NT composite alone. Therefore, the CNT ratio of CN2 (1.25 vol.%) is considered optimal. To discuss this single‐cell performance in detail, Figure S5 (Supporting Information) shows the CV curves and ECSA trends of CE and CNs. The ECSA measures the electrochemically active Pt surface area accessible to electrons and protons via the Pt/C or ionomer networks within the carbon layer. Measuring ECSA via cyclic voltammetry under an H_2_ feed at the anode and an N_2_ feed at the cathode in PEMFC is associated solely with electron and proton conduction, not with oxygen within the electrode; thus, oxygen transport is not reflected in this measurement.^[^ ^49^ ^]^ Therefore, since the total amount of catalyst (Pt/C) is identical across all samples in this study, the extent of carbon corrosion during the ADT is expected to be similar, resulting in comparable ECSA degradation among the samples. As expected, Figure S5f (Supporting Information) shows similar ECSA trends before and after ADT for CE and CNs. Therefore, the influence of the CNTs and NT composite additives in the cathode, along with other factors and measurements, must be considered. Specifically, attention should be given to mass transport improvement through the porous structure after ADT, even when the ECSA decreases similarly to CE.

**Figure 3:**
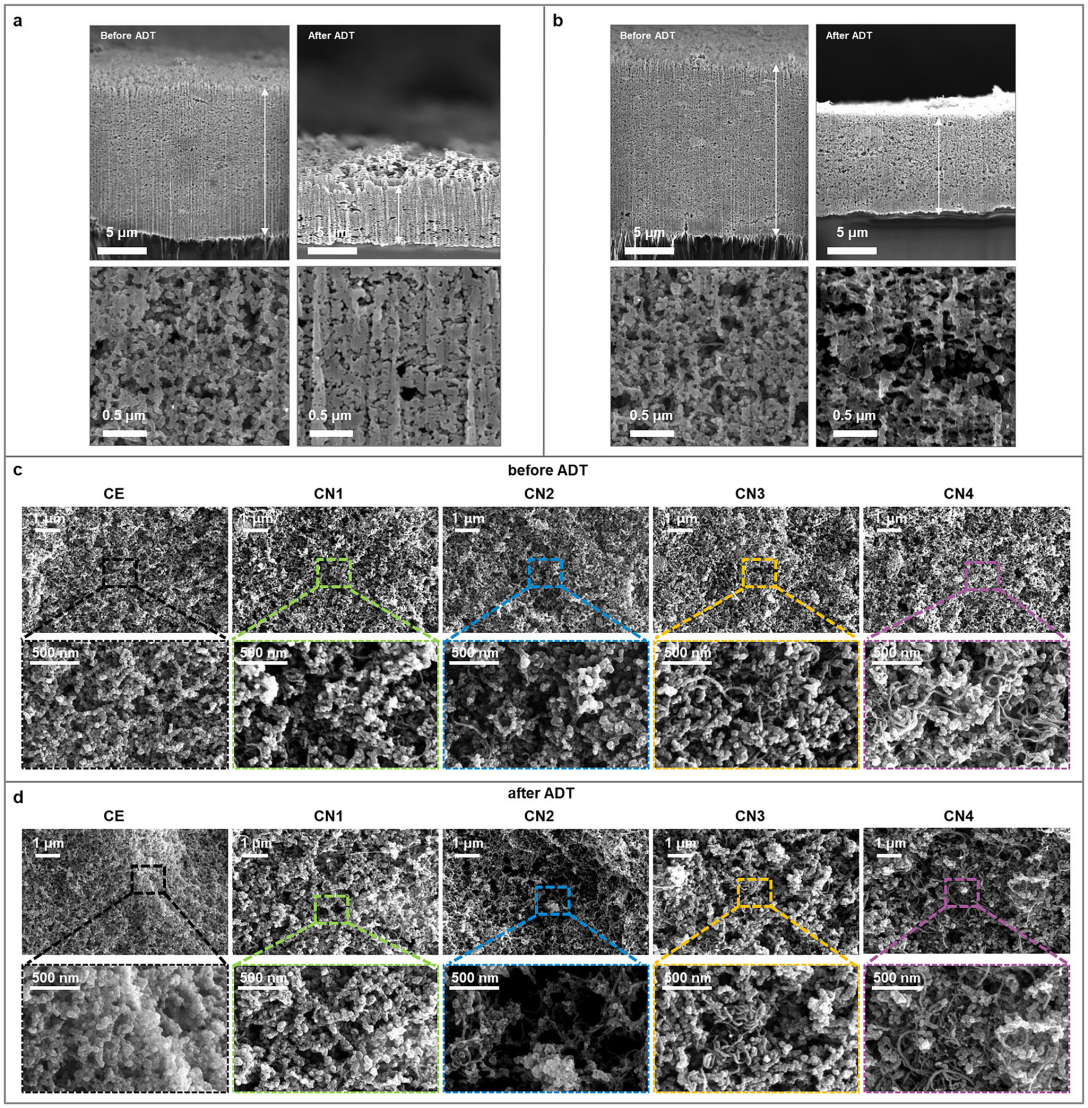
Cross‐sectional FIB‐SEM images of a) CE and b) CN2 before and after ADT and FE‐SEM images of the surface structure of the cathodes with different ratios of CNTs c) before and d) after ADT.

The CN samples were assessed by HR‐TEM with energy‐dispersive X‐ray spectroscopy (EDS) mapping to analyze the elemental composition and dispersion of the ink slurry (composed of Pt/C with CNTs and the NT composite as additives) and the electrode coating applied to the membrane using the ink slurry. Careful mixing of the NT composite with the other components of the catalyst ink, Pt/C, and CNTs, thoroughly dispersed the three components in the ink state (Figure S1, Supporting Information). The cross‐sectional structures and surface of the cathode were examined using FIB‐SEM and FE‐SEM as shown in **Figure** **3**. The cross‐sectional SEM image (Figure 3a) confirms that the thickness of the CE decreases by 67.9% after the ADT (from 13.4 to 4.3 µm). In addition, the porous structure of the CE is broken down after the ADT, accompanied by carbon loss owing to severe carbon corrosion. Compared with the CE, the CN2 coating is thicker before the ADT (Figure 3b), owing to the incorporation of the CNT and NT composite additives. By the end of the ADT, the thickness decreases by only 34.6% (from 15.9 to 10.4 µm) without structural collapse. Moreover, the CNT and NT composites not only retain the structures and porosities they had before the ADT but also reinforce the electrode structure by forming textured micrometer‐scale pores after the ADT where the carbon had been lost or where its structure had collapsed as a result of carbon corrosion.

As shown in Figure S10 (Supporting Information), cathodes using only the NT composite as an additive demonstrate an excellent effect in maintaining the porous structure after an ADT. However, unlike NT composites, which mitigate structural degradation by forming sub‐micrometer‐scale pores, CN2, with the addition of CNTs, exhibited a textured structure (with micrometer‐scale pores) after the ADT. These structural differences demonstrate the distinct effects of each NT composite and CNTs as structural additives in the cathode. These effects are also observed on the electrode surface, as shown in Figure 3c,d. The electrode surface images show that CNT, as structural additives, can generate additional micrometer‐scale pores during ADTs; in particular, CN2 exhibits a micrometer‐scale porous structure after ADTs, facilitating high mass transport through the structure. However, due to CNT agglomeration, the pore size is somewhat reduced in samples containing excessive CNT additives (CN3 and CN4). Therefore, adding only the NT composite, which forms micrometer‐scale pores after ADT, is insufficient for achieving adequate mass transport improvement. The optimal addition of both the NT composite and CNTs has demonstrated the formation of a micro‐scale pore structure after ADT.

An electrochemical analysis was conducted to further explain the function of CNTs and NT composite as structural additives. As shown in Figure S2 and Table S1 (Supporting Information), the EIS data obtained from an H_2_/air‐fed PEMFC system at 100% RH, with CE and CNs as the cathode, were fitted with a transmission line model (TLM) equivalent circuit to analyze the resistance factors in the PEMFC system. The EIS was measured at 0.85 V because it focused on analyzing the information related to the R_ion_ and R_ct_.^[^ ^50^, ^51^, ^52^ ^]^

In the case of HFR, as shown in Table S1 (Supporting Information), the HFR of CN samples increased after ADT. Furthermore, The R_ion_ is typically present in the electrochemical impedance results involving Faradaic processes.^[^ ^53^, ^54^ ^]^ However, in this study, the R_ion_ values were negligible and showed insufficient deviation to observe the effect of structural additives (CNT and NT composite). Therefore, rather than focusing on the effect of structural additives on R_ion_, changes in R_ct_ provide a more relevant explanation of the function of the additives within the context of this study.

In the initial state, CN1 and CN2 exhibit a decrease in R_ct_ compared to CE. However, the cathode with an excessive amount of the CNTs showed a higher R_ct_ than even CE. Moreover, unlike CE, the CN samples exhibit lower R_ct_ values after the ADT. A lower R_ct_ generally indicates an improvement in electrode structure and design within the PEMFC system, especially considering the use of the same catalyst (Pt/C) in all samples. Therefore, these results demonstrate that the optimal amounts of CNTs and the NT composite as structural additives effectively decrease R_ct_ both before and after the ADT by improving the electrode structure.

Two reference studies are available that support our findings that the textured electrode supports improved mass transport because of the formation of micrometer‐scale pore structures in the electrode. Ahn et al. designed an electrode with cracks and showed that the cracks can improve mass transport.^[^ ^41^, ^42^ ^]^ They controlled the crack size by using a prism‐patterned membrane to define the optimal crack size for improving mass transport, demonstrated by oxygen gain analysis. Owing to the enhanced mass transport, the optimal crack size (≈2.1 µm) improved the initial performance by ≈11% (voltage at 1.5 A cm^−2^), the largest improvement compared to the CE. This result adds value to our discussion regarding the effect of CN2 in this study, in that it supports that the formation of the micrometer‐scale pore structure (after corrosion of the carbon support by the ADT) can improve the performance such that it is higher after the ADT, in addition to improving the durability of the electrodes. In addition, Lee et al. explored the use of grooved electrodes to control the electrode structure to improve the performance by improving the mass transport of the electrode.^[^ ^43^ ^]^ They investigated the effects of the size of groove dimensions, H^+^ transport, and oxygen transport on the structurally controlled electrode. Additionally, they studied the effect of the structural collapse of the electrode due to the carbon support ADT. They showed that a groove width of 1 µm/3 µm delivered the highest performance (the voltage at 1.5 A cm^−2^ was ≈24% higher than that of the CE). In addition, they examined the durability of the grooved electrode under carbon support ADT. After 500 cycles during which the voltage was swept from 1.0 to 1.5 V, the carbon loss of the groove electrode was similar to that of the CE; however, its higher mass activity enabled superior durability. Furthermore, they investigated the effect of the ionomer content on H^+^ transport. Variation of the ionomer/carbon ratio from 0.9 to 1.2 resulted in a 60% decrease in the H^+^ transport resistance at 100% relative humidity. For the groove electrode, they mentioned that the uniform distribution of ionomer and Pt is important for controlling the electrode morphology. These two approaches to control the electrode structure support our electrode design, which results in the formation of micrometer‐scale pore structure during the ADT. This prompted us to design a grooved electrode with well‐dispersed components (Pt/C, ionomer, CNTs, and NT composite, Figure S1, Supporting Information) and the ability to maintain its structure with micrometer‐scale pores without any part of their collapsing. Particularly in the case of CN2 (Figure 3d), even though the pores did not have equal sizes, the micrometer scale pathway was sufficient to improve mass transport. Furthermore, one of our additives (the NT composite), fabricated by coating the non‐corrosive material TiO_2_ with Nafion ionomer, can serve as an H^+^ pathway before and after ADT. The CN samples, incorporating CNTs and the NT composite as structural additives within a conventional Pt/C electrode, can improve durability after the ADT by mitigating the structural collapse of the electrode. In addition, these electrodes demonstrated micrometer‐scale pore structures (especially in CN2) that were controlled by adjusting the CNT ratio in the cathode.

The improvement of the mass transport was further assessed by overpotential analysis, conducted to elucidate the polarization sources by measuring the resistance in the high‐frequency region using EIS (Figure S2, Supporting Information). The overpotentials derived from the ohmic, kinetic, and mass‐transfer losses of each electrode before and after the ADT are displayed in Figure S4 (Supporting Information) and explained in detail in Note S1 (Supporting Information).

For the ohmic overpotential, Spernjak et al. reported that carbon corrosion increases the contact resistance between the GDL and the catalyst layer, leading to elevated HFR after ADT.^[^ ^55^ ^]^ Consistent with their findings, all samples in this study exhibited a similar increase in HFR after ADT (Table S1, Supporting Information), which can be attributed to the use of the same amount of Pt/C in all electrodes, likely resulting in comparable levels of carbon corrosion. These results show a similar trend to the observed changes in ohmic overpotential.

In the case of kinetic overpotential, it involves not only electrons and protons but also oxygen; thus, oxygen transport plays a critical role in this method.^[^ ^56^, ^57^, ^58^ ^]^ Neyerlin et al. investigated the correlation between catalyst utilization and thickness which is not considerable in the ECSA analysis.^[^ ^57^ ^]^ Catalyst utilization decreases with increasing sheet resistance, which is typically associated with higher proton conduction resistance, often due to increased electrode thickness or reduced ionomer content per volume. Moreover, Schneider et al. reported that thicker electrodes, even with the same Pt loading, exhibited lower specific activity due to transport limitations.^[^ ^56^ ^]^ They also noted that ECSA does not always reflect actual operating performance, as specific activity can be hindered by limited oxygen or proton accessibility within the electrode structure.

In this study, CE samples showed reductions in both electrode thickness and porosity after ADT, resulting in increased kinetic overpotential consistent with ECSA loss. In contrast, CN samples initially exhibited higher thickness and lower porosity due to increased CNT content (especially for CN3 and CN4), which likely led to greater sheet resistance and higher initial kinetic overpotential. However, after ADT, the CN electrodes became thinner while maintaining their porous structure, reducing sheet resistance and improving oxygen accessibility.^[^ ^56^, ^57^ ^]^ This structural preservation likely enhanced catalyst utilization and explains the observed decrease in kinetic overpotential despite continued ECSA loss.

**Figure 4:**
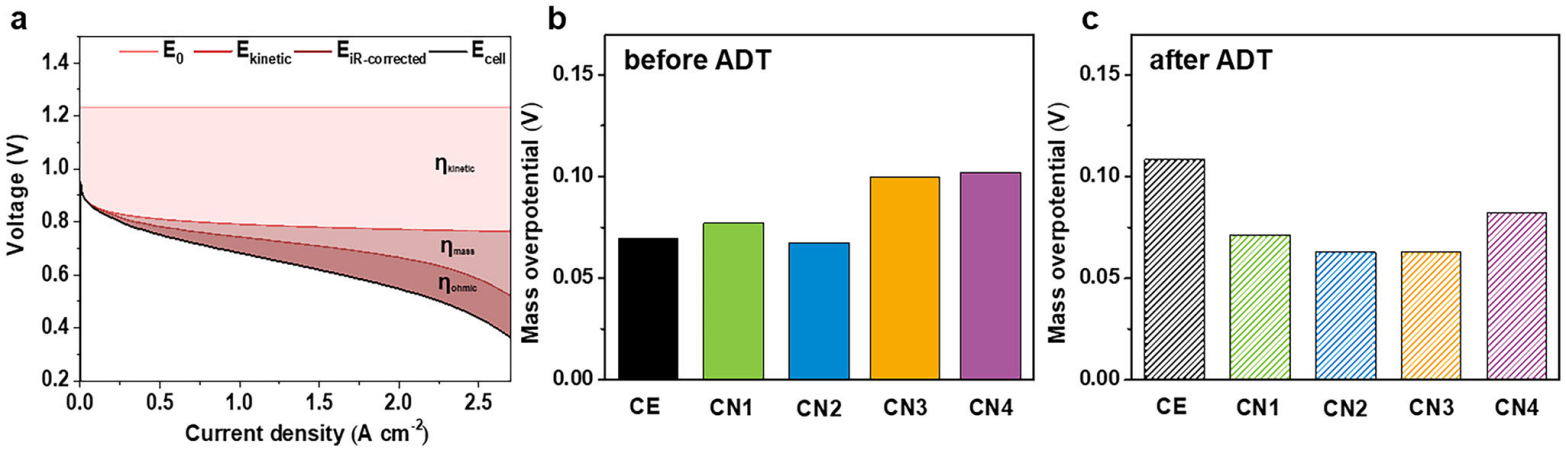
Voltage breakdown of polarization curve based on the overpotential analysis with the three main losses. Mass overpotential analysis at a fixed current density of 1.5 A cm^−2^ (b) before ADT and (c) after ADT.

Furthermore, the mass overpotential (**Figure** **4**a,b) of the electrodes before the ADT is similar for the CE, CN1, and CN2, regardless of the addition of the CNTs and NT composite, but it is inhibited by the excess amount of CNTs in CN3 and CN4. Although the thickness of the cathode increases (Figure S6, Supporting Information) from adding the CNTs, the pore structures of CN1 and CN2 are similar to that of the CE before the ADT (Figure 3). Furthermore, considering both the mass overpotential and the PEMFC performance, CN2 has the optimal composition before the ADT (Figure 4b). Additionally, the results after the ADT show that the effects of adding the CNTs and NT composite are significant. Although the mass overpotential of the CE increases by 56.1% (from 0.069 to 0.108 V), those of the CN samples decrease considerably during the ADT; notably, the mass overpotential of CN2 decreases by ≈7.4% (from 0.068 to 0.063 V). This difference in the mass overpotential can be explained by the porosity and thickness of the cathode.^[^ ^59^ ^]^ To demonstrate the function of CNT in this study, Figure S11 (Supporting Information) presents the overpotential analysis before and after an ADT for the NT composite‐only sample. The mass transport overpotential of the cathode using the NT composite as the sole additive increased by 23.9% (from 0.071 to 0.088 V) after the ADT. While this is still effective in reducing mass transport overpotential than CE, it remains insufficient. Therefore, unlike the mass transport overpotential of CE and the NT composite‐only sample, which increased after an ADT, the mass transport overpotential decreased in the presence of CNTs after an ADT.

In addition, this overpotential analysis shows that the micrometer‐scale pores formed in CN2 during the ADT strongly affect the mass transport owing to the high oxygen accessibility at the triple‐phase boundary on the cathode both before and after the ADT. Therefore, among the electrodes to which the CNTs and NT composite have been added, CN2 proves to have the optimal composition, with both high performance and durability, and these results are related to the mass overpotentials. The overpotential analysis to determine the effect of the structural additives in the cathode indicates that the mass overpotential is the major factor responsible for determining the PEMFC performance. Thus, the substantial improvement in the performance and durability of CN2 can be ascribed to the retention of its suitable micrometer‐scale pore structure with optimal thickness for the duration of the ADT, together with its optimal ratio of CNTs to NT composite. Apart from this, optimizing the pore size and structure of the electrode and stabilizing it even under severe carbon corrosion can be an effective strategy not only for achieving a structure that is highly sustainable until after the ADT, but also for the practical commercialization of PEMFCs.

The effect of CNT and the NT composite as structural additives can also be demonstrated by considering mass transport resistance and oxygen diffusion through an oxygen gain analysis. This analysis was performed to ascertain whether the micrometer‐scale pore structure resulting from the addition of the CNT and NT composite could enhance the durability by improving the mass transport in electrodes, leading to better oxygen diffusion and reduced oxygen transport resistance in the CNs. An increase in the partial pressure of oxygen in the cathode could be expected to slightly increase the voltage of a cathode with highly developed gas transfer channels, whereas that with an insufficient pore network would be expected to show a larger increase in voltage.^[^ ^60^ ^]^ Thus, a lower oxygen gain implies the more facile transport of gases through the well‐developed porous catalyst layer and also a lower mass‐transfer resistance.^[^ ^61^ ^]^ The oxygen gain is calculated using the following equation:

(1) $$\mathrm{Oxygen}\;\mathrm{gain}=V_{O_{2}}\;-V_{\mathrm{air}}$$

**Figure 5:**
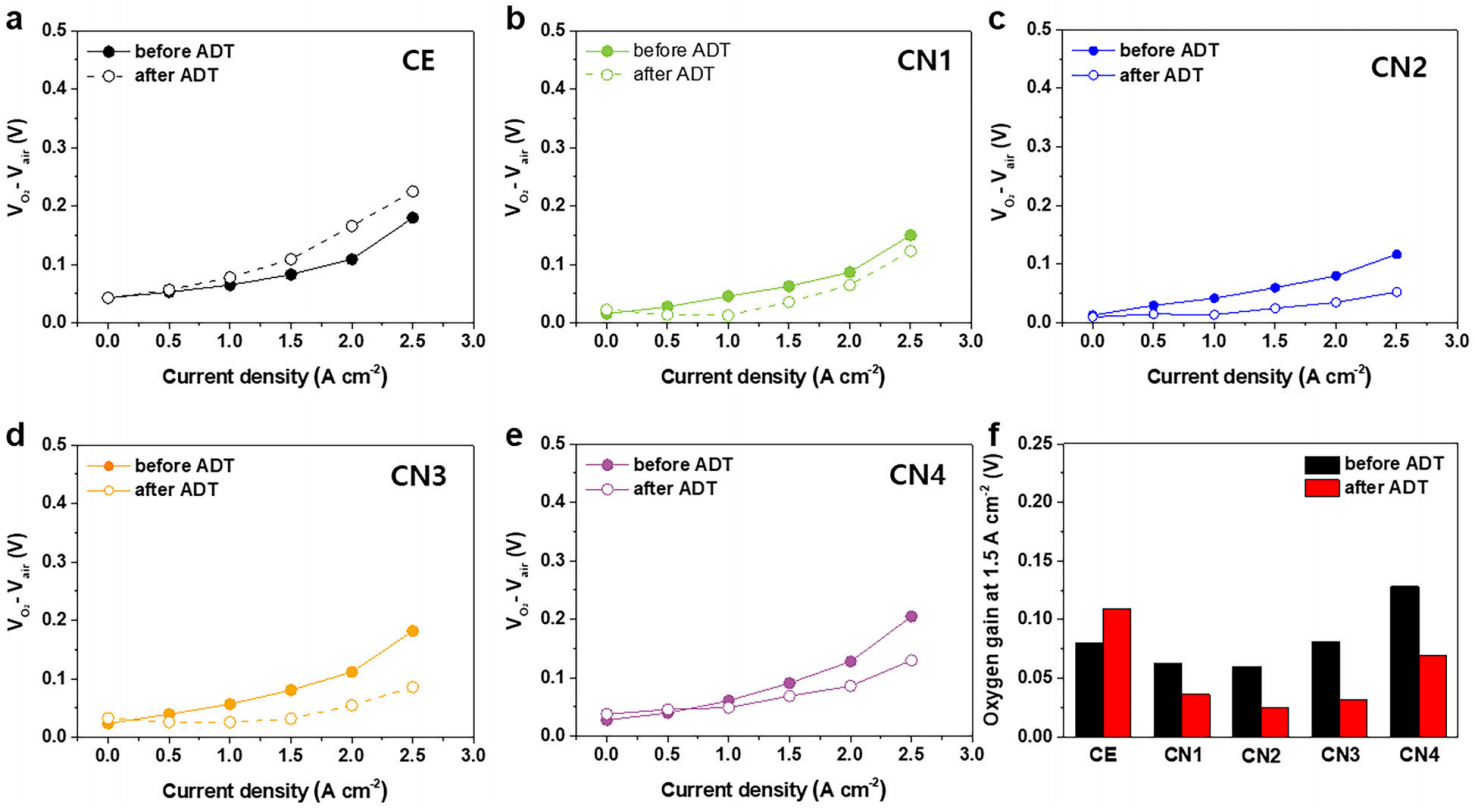
Oxygen gains for MEAs with a) CE, b) CN1, c) CN2, d) CN3, and e) CN4; f) comparison of the oxygen gain (at 1.5 A cm^−2^) of the different MEAs.

The oxygen gains of the electrodes were determined by recording the polarization curves of the cathode in both air and oxygen, pressurized at 1.0 bar. The oxygen gains of the MEAs were compared at a current density of 1.5 A cm^−2^; the results are shown in **Figure** **5**. In the initial state, as shown in Figure 5f, the oxygen gains of both CN1 and CN2 are lower than that of the CE, whereas CN3 and CN4 exhibit higher oxygen gains at 1.5 A cm^−2^. These results indicate that an optimal amount of CNTs not only improves the porous structure before the ADT but also facilitates mass transfer through the textured micrometer‐scale pores that form during the ADT. Figure S12 (Supporting Information) shows the oxygen gain of the cathode using NT composite as the sole additive. The NT composite decreased the oxygen gain after ADT; however, it exhibited a similar oxygen gain to CN2 before ADT. These results indicate that using CNT with NT composite can further decrease the oxygen gain both before and after ADT, particularly after ADT due to the formation of a textured micro‐scale pore structure with an optimal amount of CNT. However, entangled, aggregated excess CNTs can block the pores inside the cathode, thereby impeding the transport of the gaseous feedstock and liquid products. Among the CNs, CN2 exhibits the lowest oxygen gain, based on its prominently improved behavior in the mass‐transfer region, thereby resulting in the highest initial performance. Furthermore, the sharp increase in the oxygen gain of the CE in the mass transfer–dominating region (+36% at 1.5 A cm^−2^) measured after the ADT, is ascribed to the collapsed porous structure as a result of carbon corrosion. In contrast, the oxygen gains of the CN samples decrease (−60% to −43% at 1.5 A cm^−2^); consequently, the mass transfer of the CN samples considerably improves during the ADT. This facilitated mass transfer can be understood in terms of the alleviation of structural collapse and the formation of porous structures (particularly the micrometer‐scale pores in CN2), resulting from the significant effect of the CNTs and NT composite to combat carbon corrosion. The fact that CN2 has the lowest oxygen gain both before and after the ADT demonstrates that it has the most effective structure for supporting facile mass transfer with an optimal CNT ratio as a result of the formation of a micrometer‐scale pore structure during carbon corrosion.

This study demonstrates a practical strategy for enhancing MEA durability by using commercial Pt/C, eliminating the need for additional synthesis to replace the catalyst support. Furthermore, as shown in Figure S13 and Note S2 (Supporting Information), the effectiveness of using CNT and NT composites is highlighted in achieving both high durability and performance without compromise by comparing other studies.^[^ ^15^, ^17^, ^21^, ^31^, ^32^, ^62^ ^]^

In future work, we will investigate the detailed mechanisms and structural characteristics of the electrode that govern carbon corrosion and structural collapse, including under more severe ADT conditions, with the aim of improving durability while maintaining high initial performance.

## 3 Conclusion

In this study, we introduced a new concept in the form of an advanced electrode that incorporated CNTs (for textured micrometer‐scale pores) and the NT composite (for reinforced sub‐micrometer‐scale pores) as a strategy to improve the durability of the PEMFC system. The CNTs and NT composite (which consisted of ionomer‐coated inorganic nanoparticles) were simply applied to Pt/C via physical mixing and auto‐spraying. In the optimal ratio, the CNTs and NT composite served as structural supports and provided additional ionic channels within the electrode to simultaneously improve the durability of the PEMFC system by forming a micrometer‐scale pore structure in the cathode as a result of carbon corrosion. CN2 exhibited the highest voltage (0.63 V at 1.5 A cm^−2^) in the initial state and the lowest performance degradation of 0.3% after the ADT (the durability was ≈37 times higher than that of the CE). Although the voltage initially exhibited by the CE (0.62 V) was similar to that of CN2, the performance degradation that occurred during the ADT exceeded 11%. The effects of the incorporation of the CNTs and NT composite as additives were analyzed. These additives led to the development of a porous structure during PEMFC operation, thereby increasing the mass transport measured after the ADT by forming a micrometer‐scale porous structure. Structural analysis using SEM confirmed that the advanced porous structure of CN2 remained intact even after severe carbon corrosion because the additives (CNTs and NT composite) effected in preventing the structural collapse that generally occurs in the CE. Therefore, the concept of enhancing the cathode with simple additives (the CNTs and NT composite in an optimal ratio) can be considered a promising strategy to improve electrode durability and structural reinforcement in PEMFC systems under carbon corrosion conditions. Additionally, it facilitates the commercialization of PEMFCs by enabling the continued use of the conventional catalyst.

## 4 Experimental Section

### MEA Fabrication

All the MEAs in this study contained identical anodes, which comprised a commercial Pt/C catalyst (46.5 wt.% Pt, Tanaka Co.) with 0.2 mg_Pt_ cm^−2^ loading. The ink used to fabricate the anode catalyst was prepared by ultrasonically mixing a commercial Pt/C catalyst with Nafion ionomer (5 wt.% solutions, Sigma‐Aldrich Co.) in isopropyl alcohol (IPA) (Daejung Co.) at an ionomer/carbon ratio of 0.7. The well‐mixed catalyst ink was auto‐sprayed onto one side of a Nafion 211 membrane.

However, the composition of the cathode of each MEA was different. Enhanced MEAs were fabricated by simultaneously applying CNTs (multi‐wall CNTs, Sigma‐Aldrich Co.) and NT composites to the cathode side. The NT composite was synthesized by coating commercial TiO_2_ nanoparticles (Aeroxide P25, Evonik) with the Nafion ionomer at a volumetric ratio of 1:0.8 (TiO_2_:Nafion). TiO_2_ powder was dispersed in IPA using ultrasonication for 10 min. The pH of the TiO_2_‐IPA suspension was carefully adjusted to between two and three by adding 1 m HClO_4_ to ensure uniform dispersion. 25% of the total Nafion ionomer (intended for the final electrode) was separately dispersed in IPA via ultrasonication for 10 min and subsequently added to the TiO_2_ suspension. The resulting mixture was further mixed by ultrasonication for 30 min to facilitate the coating of the individual TiO_2_ particles by the Nafion ionomer while maintaining high dispersity. The resulting NT composite was dried overnight in a vacuum oven at 80 °C. Further details on the synthesis are provided in our previous report.^[^ ^45^ ^]^

In addition to the NT composite, CNTs were used as composite supplements. The volumetric ratio of the CNTs was varied from 0.5 to 5.0 with reference to the volume of the Pt/C catalyst, whereas the as‐prepared NT composite was applied to each MEA at a fixed volumetric ratio of 6.25 with reference to the Pt/C catalyst. To prepare the ink containing the cathode catalyst for application to the MEA, the CNTs and NT composite were dispersed individually in IPA by vigorously stirring the mixture for 30 min, followed by ultrasonication for 30 min. The commercial Pt/C catalyst was ultrasonically mixed with 5 wt.% Nafion ionomer in IPA for 30 min, whereupon all the solutions were combined and ultrasonicated for 30 min. The cathode catalyst inks with different CNT ratios were auto‐sprayed (120‐kHz ultrasonic nozzle assembly, SONOTEK) on the reverse side of the Nafion membrane to form cathodes with a Pt loading of 0.4 mg cm^−2^.

The thickness of the cathode on each MEA differed owing to the varied composition; hence, gaskets with different thicknesses were applied to the cathode side of the MEAs to ensure that all the cathodes experienced the same level of compression of ≈1.4% (Figure S5, Supporting Information).

### Characterization of Electrochemical Properties

The electrochemical characteristics of the MEAs were evaluated using a single‐cell system. Polarization curves were recorded to examine the PEMFC performance before and after the durability test of each MEA. During the measurements, the gases that were supplied to the anode (H_2_) and cathode (O_2_ or air) at flow rates of 200 and 600 mL min^−1^, respectively, were 100% humidified. The cell temperature was set to 80 °C with a backing pressure of 1.0 bar.

The cell was activated using the activation protocol: 0.9–0.4 V with a scan rate of 20 mV s^−1^, and 3 h hold at 0.6 V under H_2_/O_2_‐fed. After the polarization test, electrochemical impedance spectroscopy (EIS) measurements were performed using a potentiostat (SP‐300, BioLogic) at an applied voltage of 0.85 V in the frequency range of 10 kHz to 100 mHz. Fit was generated using the ZView software. Cyclic voltammetry (CV) curves were recorded in the voltage range of 0.05–1.2 V at a scan rate of 50 mV s^−1^ to calculate the electrochemically active surface area of the catalyst. Furthermore, after the ADT, the EIS and CV profiles were acquired under conditions identical to those before the ADT to assess the durability of the PEMFC system.

### Accelerated Durability Test (ADT)

The anode and cathode were exposed to humidified H_2_ and humidified N_2_ at flow rates of 200 and 600 mL min^−1^, respectively. To force the cathode catalyst to undergo carbon corrosion, the ADT was conducted by applying a constant voltage of 1.3 V to the cell for 10 h under ambient pressure. Among the various ADT methods for carbon corrosion, applying a constant voltage of 1.3 V has been extensively studied.^[^ ^55^, ^63^ ^]^ Spernjak et al. reported that modifying the ADT protocol to a 1.3 V hold accelerated carbon corrosion under harsher conditions compared to the standard carbon corrosion ADT at a 1.2 V hold.^[^ ^55^ ^]^ Furthermore, following a standard carbon corrosion ADT with voltage cycling (1.0–1.5 V), as outlined in the U.S. DOE protocol,^[^ ^6^ ^]^ similar performance degradation and reductions in cathode thickness were observed after a 10‐h hold at a constant 1.3 V hold and after 5000 voltage cycles between 1.0 and 1.5 V, as shown in Figures S7 and S8 (Supporting Information).

### Physical Characterization

The morphologies of the synthesized NT composite and the catalyst ink containing the composite and CNTs were confirmed using high‐resolution transmission electron microscopy (HR‐TEM, Talos F200X, Thermo Fisher Scientific). Additional analyses of the surface and cross‐sectional structural properties of the prepared cathodes were performed using field‐emission scanning electron microscopy (FE‐SEM, Teneo VS, FEI) and focused ion beam scanning electron microscopy (FIB‐SEM, Helios NanoLab 600, FEI).

## Conflict of Interest

The authors declare no conflict of interest.

## Supporting information



Supporting Information

## Data Availability

The data that support the findings in this study are available from the corresponding author upon reasonable request.
