# Supplementary material for: Electrode Structuring via Carbon Nanotubes and Nafion Ionomer–Coated TiO2 Enhances the Durability of Proton Exchange Membrane Fuel Cells Under Carbon Corrosion Conditions
Source: Small. 2025 Mar 25;21(33):2409650. doi: 10.1002/smll.202409650 (PMC12372429; doi:10.1002/smll.202409650)
Supplement: Supplementary file 1 — Supporting Information [file SMLL-21-2409650-s001.docx]

Supporting information

Electrode structuring via carbon nanotubes and Nafion ionomer–coated TiO_2_ enhances the durability of proton exchange membrane fuel cells under carbon corrosion conditions

Ohsub Kim‡^ab^, Katie Heeyum Lim‡^a^, JunHwa Kwon ^a^, Sung Jong Yoo^abc^, Jin Young Kim^abc^, Sung Ki Cho^a^, Hyun S. Park^ab^, So Young Lee^a^, Bora Seo^ab^, Myeong-Geun Kim ^a^, Jong Hyun Jang*^abc^, Hee-Young Park*^a^

^a.^ Center for Hydrogen and Fuel Cells, Korea Institute of Science and Technology (KIST), Seoul 02792, Republic of Korea

^b.^ Division of Energy & Environment Technology, KIST School, University of Science and Technology (UST), Seoul 02792, Republic of Korea

^c.^ Green School, Korea University, Seoul 02841, Republic of Korea

‡ These authors contributed equally to this paper.

* Corresponding authors.

E-mail: jhjang@kist.re.kr (J. Jang), parkhy@kist.re.kr (H. Park).


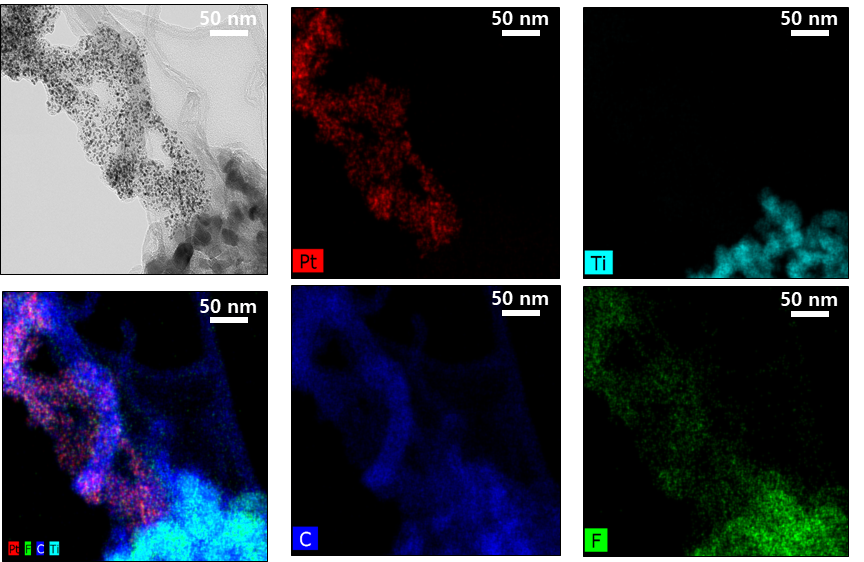


**Figure S1.** TEM images of the Pt/C catalyst ink slurry containing CNTs and Nafion ionomer–coated TiO_2_ (CN4). HR-TEM and EDS mapping.

**
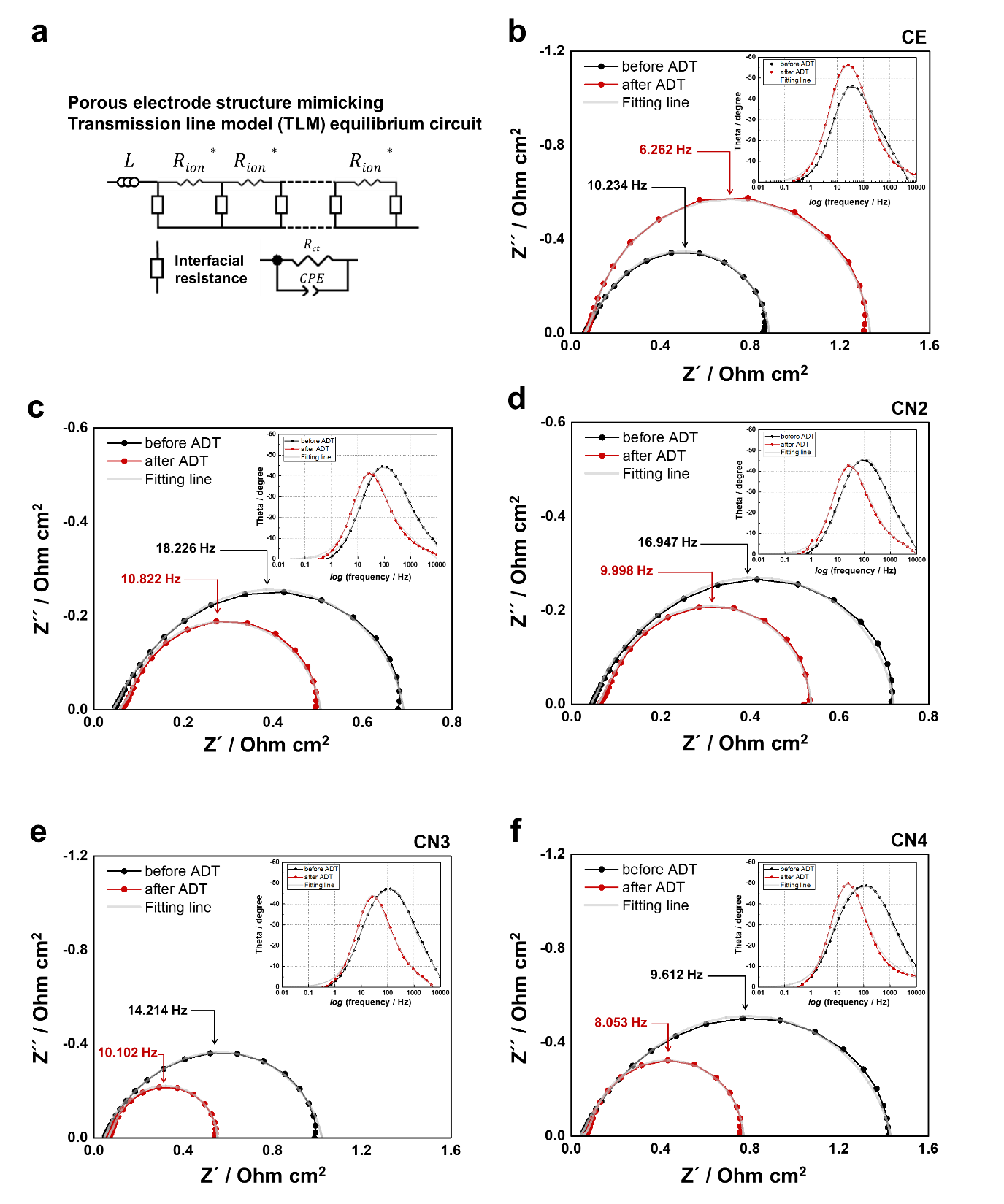
 Figure S2.** Electrochemical impedance spectroscopy (EIS) plots (a-f) with (a) TLM equilibrium circuit under 100% relative humidity of H2/air-fed PEMFC system. (b) CE, (c) CN1, (d) CN2, (e) CN3, and (f) CN4.


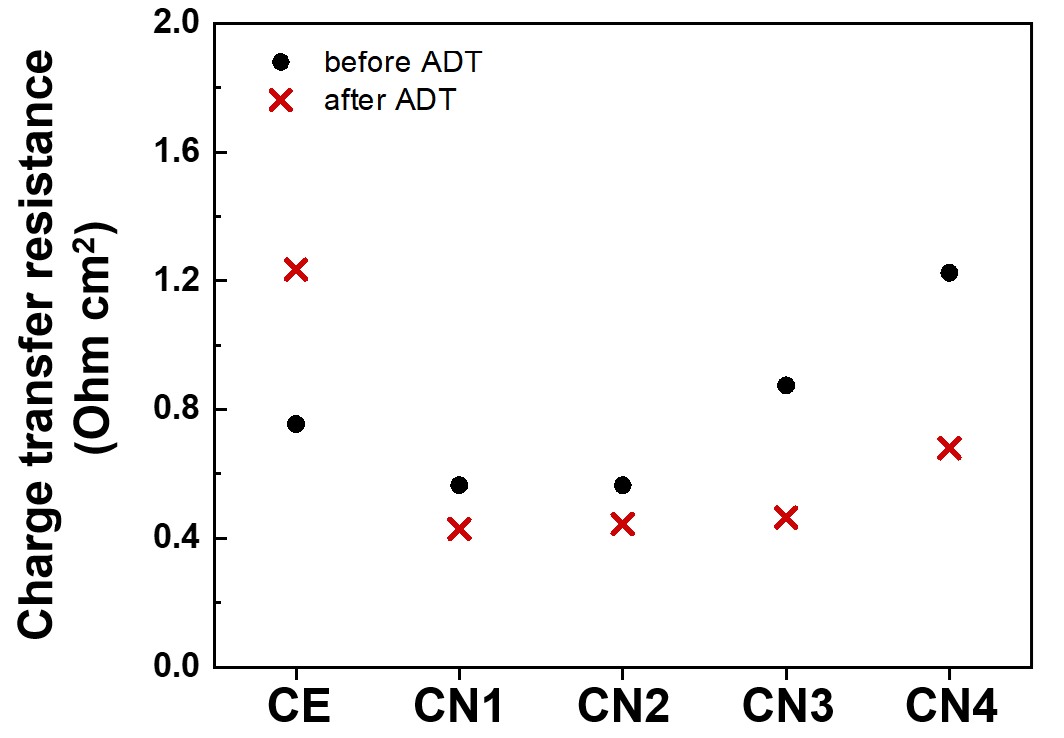


**Figure S3.** Electrochemical measurements results of charge transfer resistance trend under 100% relative humidity in a H_2_/air-fed PEMFC system.


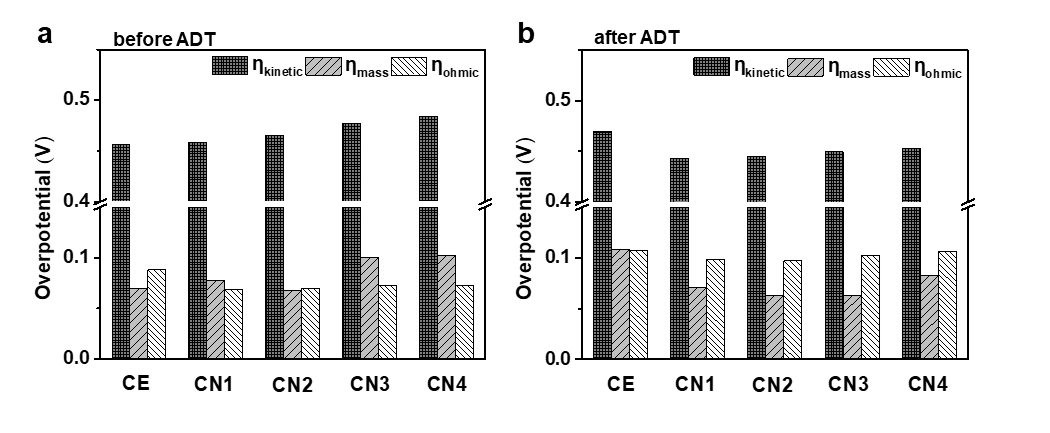


**Figure S4.** Overpotential analysis at fixed current density of 1.5 A cm^−2^ for each MEA (a) before ADT and (b) after ADT.


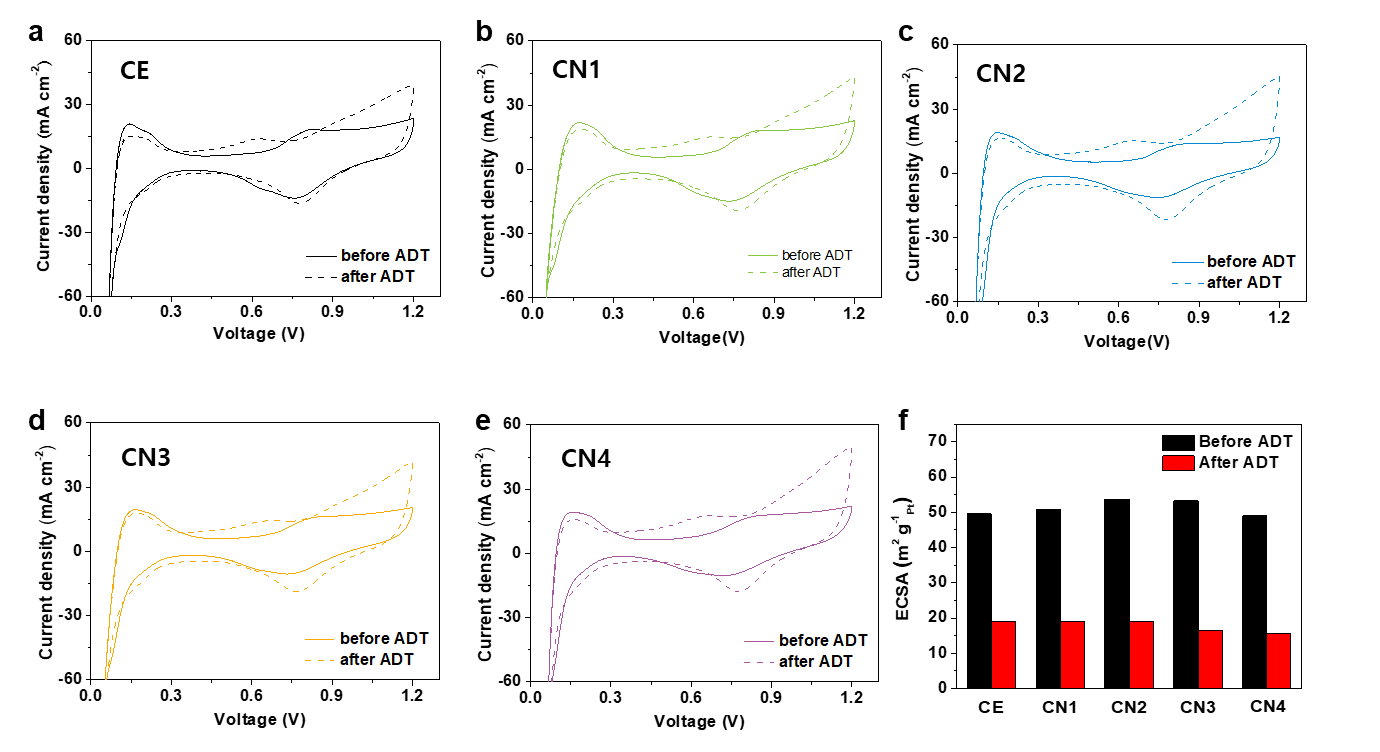

**Figure S5.** CV curves of MEAs with (a) CE, (b) CN1, (c) CN2, (d) CN3, and (e) CN4 for the cathode; (f) trend of ECSA for each MEA.


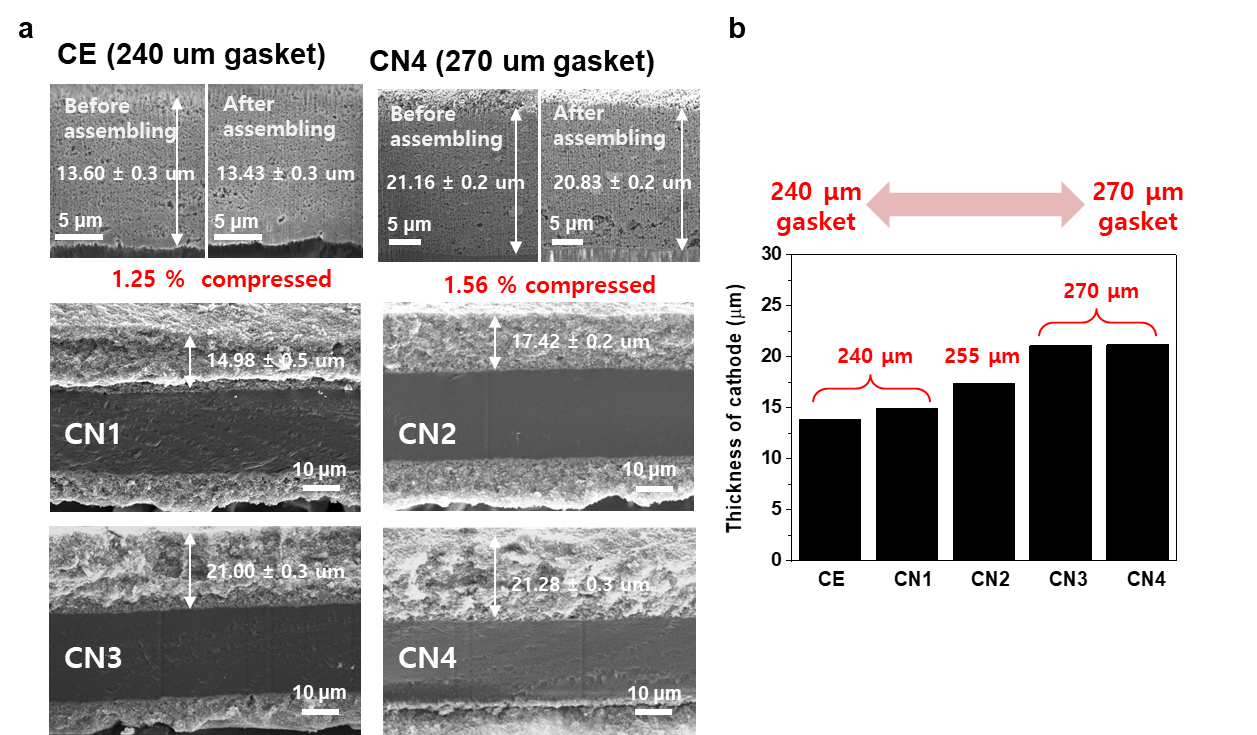


**Figure S6.** (a) Cross-sectional SEM images of electrode compressibility and (b) thickness of cathode and gasket thickness of each sample.

**
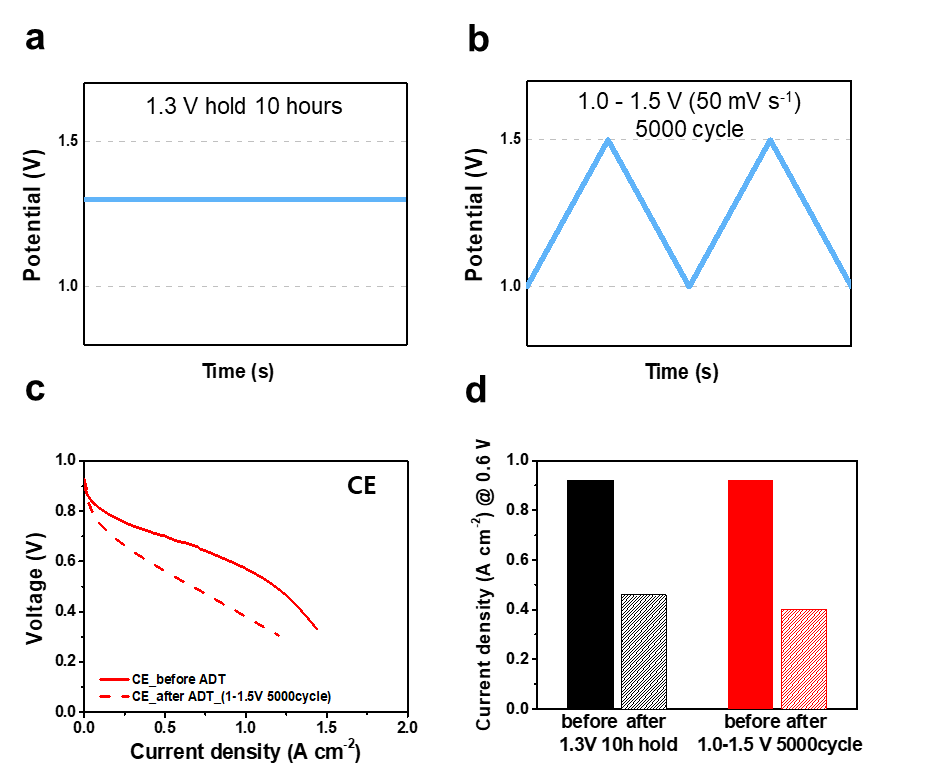
**

**Figure S7.** Single-cell performance and performance loss under different ADT protocols using the CE cathode: (a, b) ADT protocols for carbon corrosion in MEA used in (a) this study and (b) the DOE catalyst support ADT. (c) Polarization curves of CE under the DOE catalyst support ADT and (d) current density at 0.6 V under different ADT protocols.

**
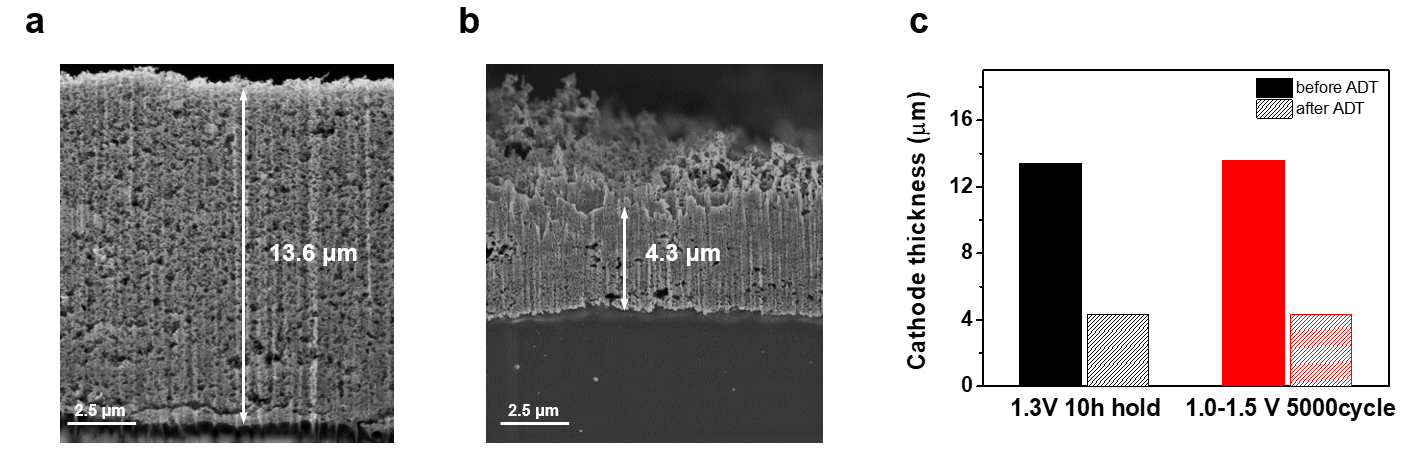
**

**Figure S8.** SEM images of cathode and cathode thickness changes under different ADT protocols: (a, b) SEM image of the cathode (a) before and (b) after the DOE catalyst support ADT (1.0–1.5 V, 5000 cycle). (c) Cathode thickness changes under different ADT protocols.

**
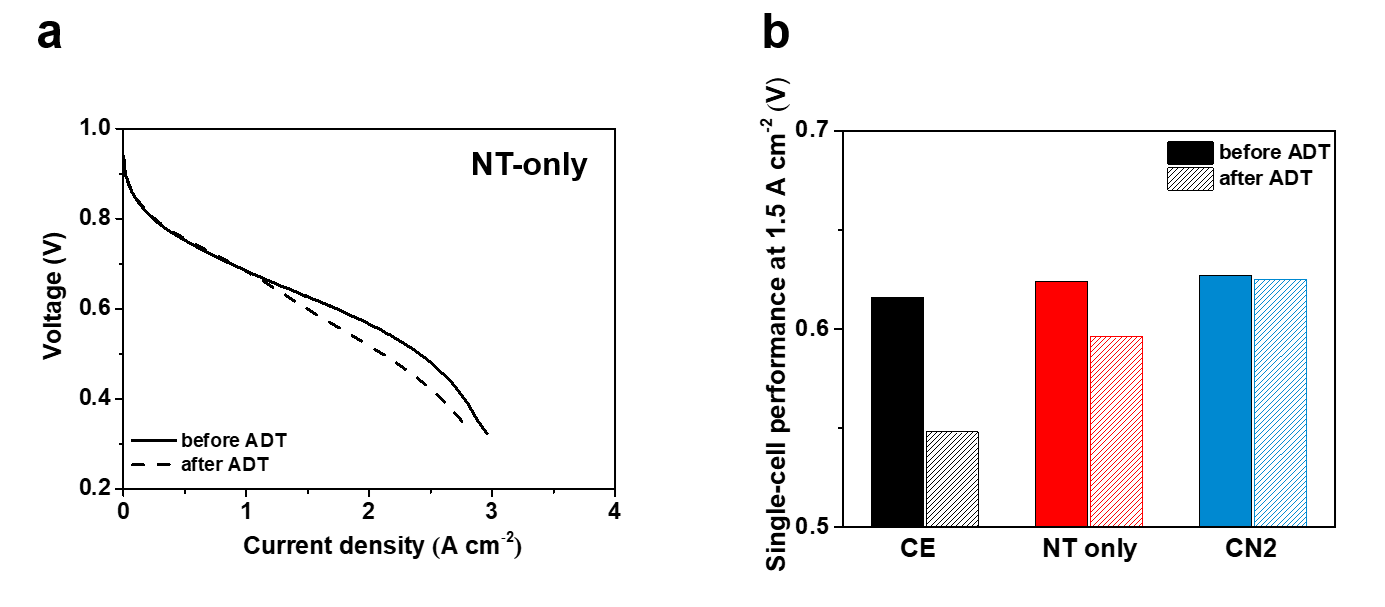
**

**Figure S9.** Polarization curves of a cathode that uses only the NT composite as an additive and (b) single-cell performance trends (at 1.5 A cm^-2^) for CE, NT only, and CN2 before and after ADT.


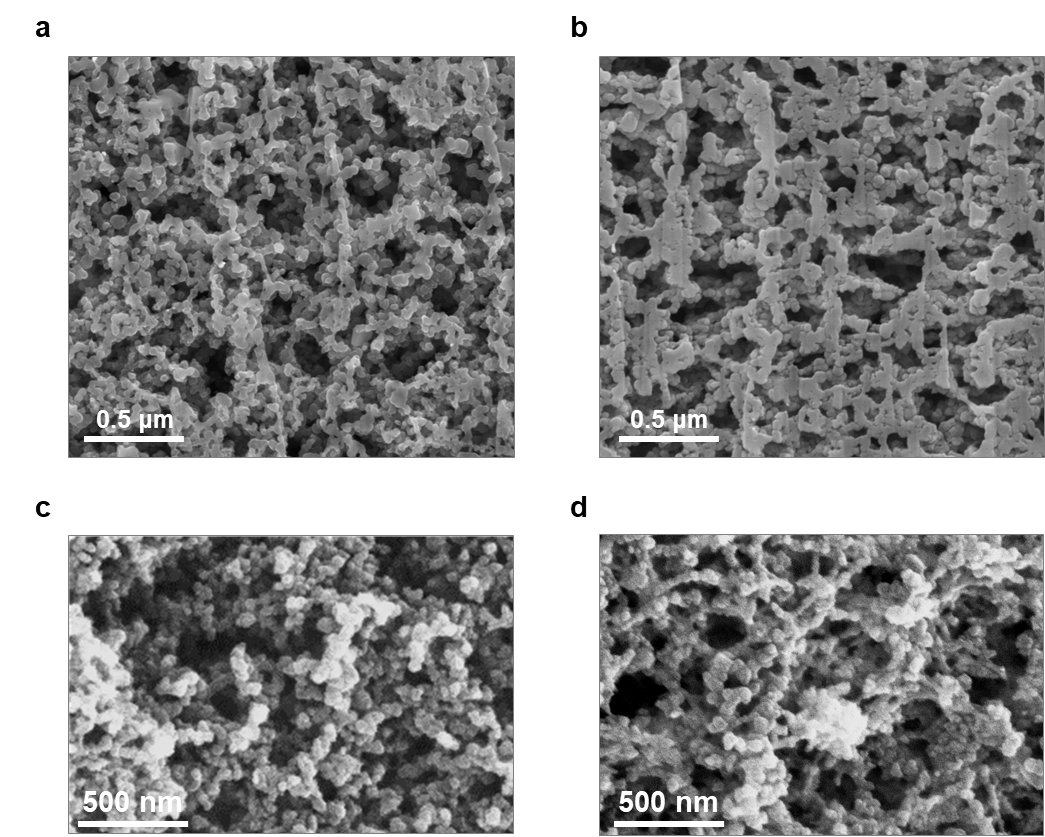


**Figure S10.** Cross-sectional FIB-SEM images of the cathode (a) before and (b) after ADT. FE-SEM images of the cathode (c) before and (d) after ADT, using only NT composite as an additive**.**


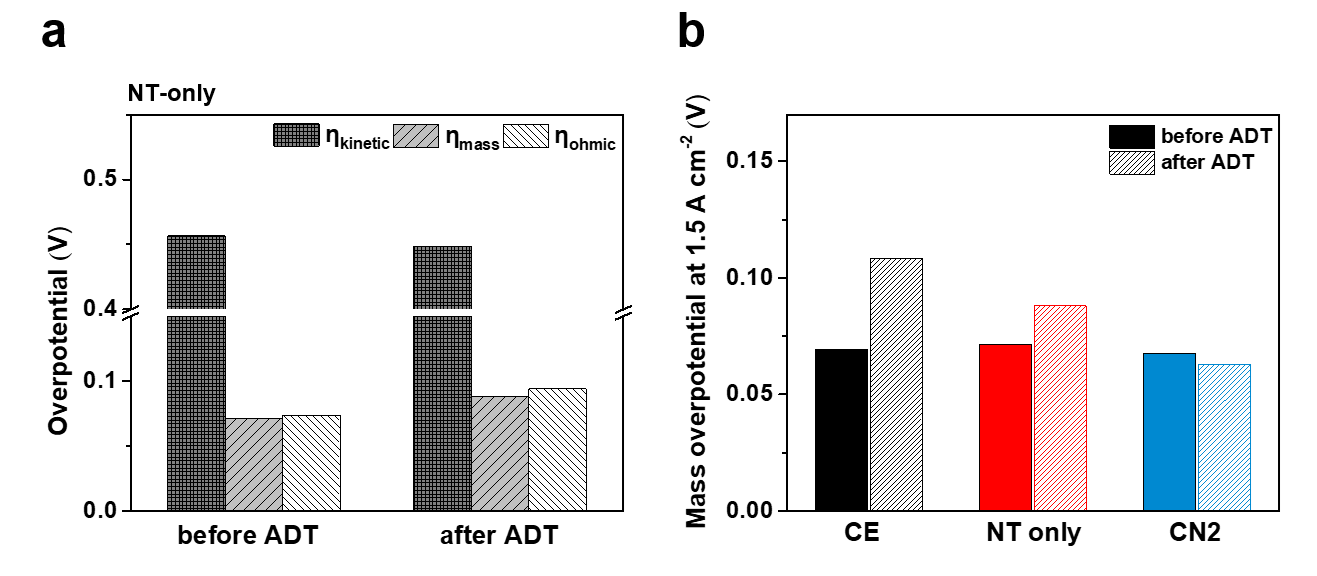


**Figure S11.** (a) Overpotential analysis before and after ADT for the cathode using only NT composite as an additive and (b) overpotential trends (at 1.5 A cm^-2^) for CE, NT only, and CN2 before and after ADT.


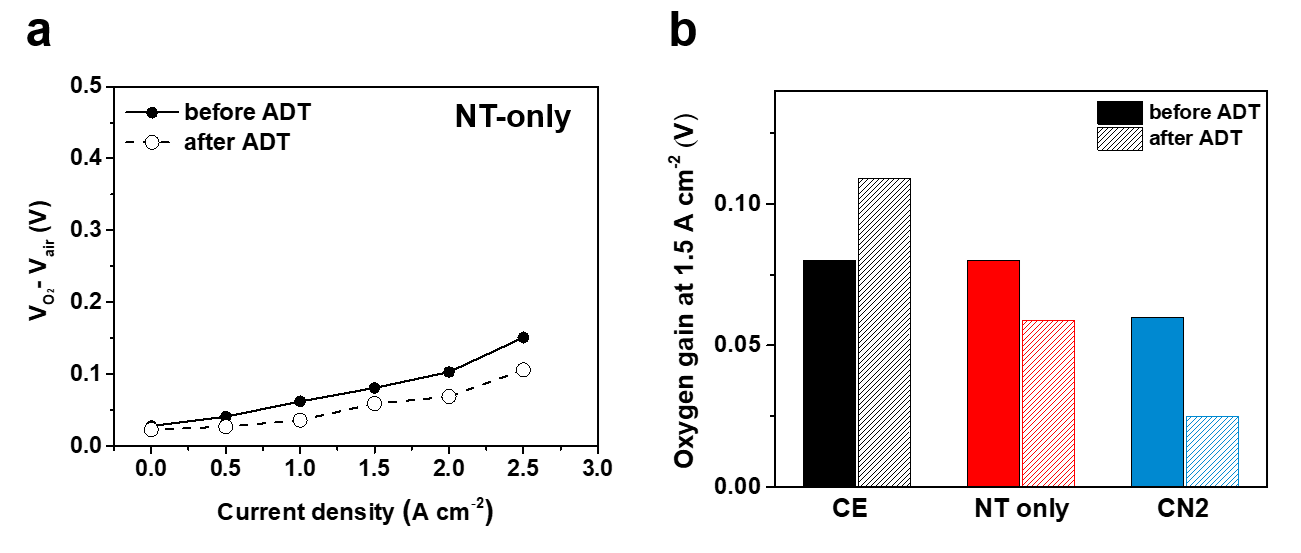


**Figure S12.** (a) Oxygen gain for cathode that using only NT composite as an additive before and after ADT and (b) oxygen gain trends (at 1.5 A cm^-2^) for CE, NT only, and CN2 before and after ADT.


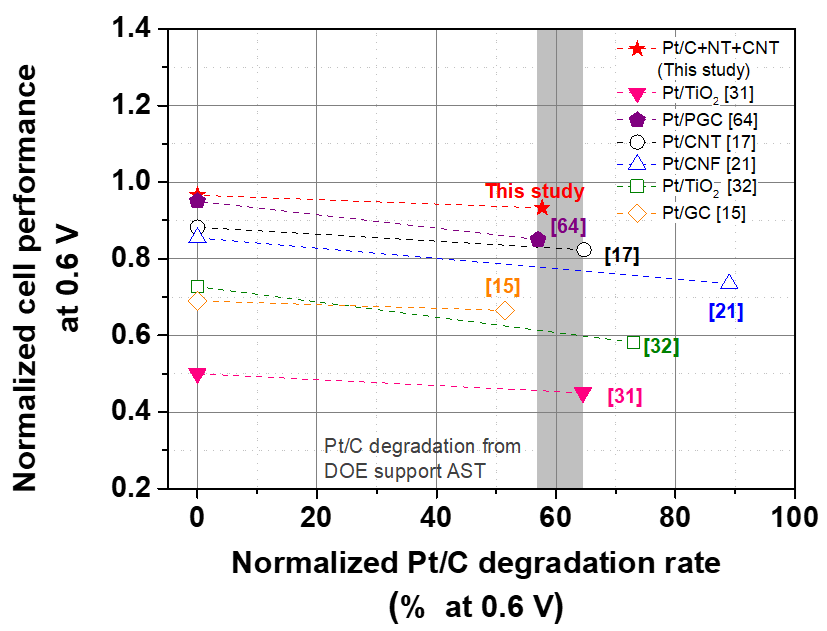


**Figure S13.** Comparison of normalized cell performance degradation of CN2 with previously reported cathodes. Ratio between EOL of CN2 at 1 bar and developed electrode to BOL of reference Pt/C electrode with a function of performance degradation rate of reference Pt/C electrode.

**Table S1.** Result of the TLM equilibrium circuit model fit for CE and CNs before and after ADT.


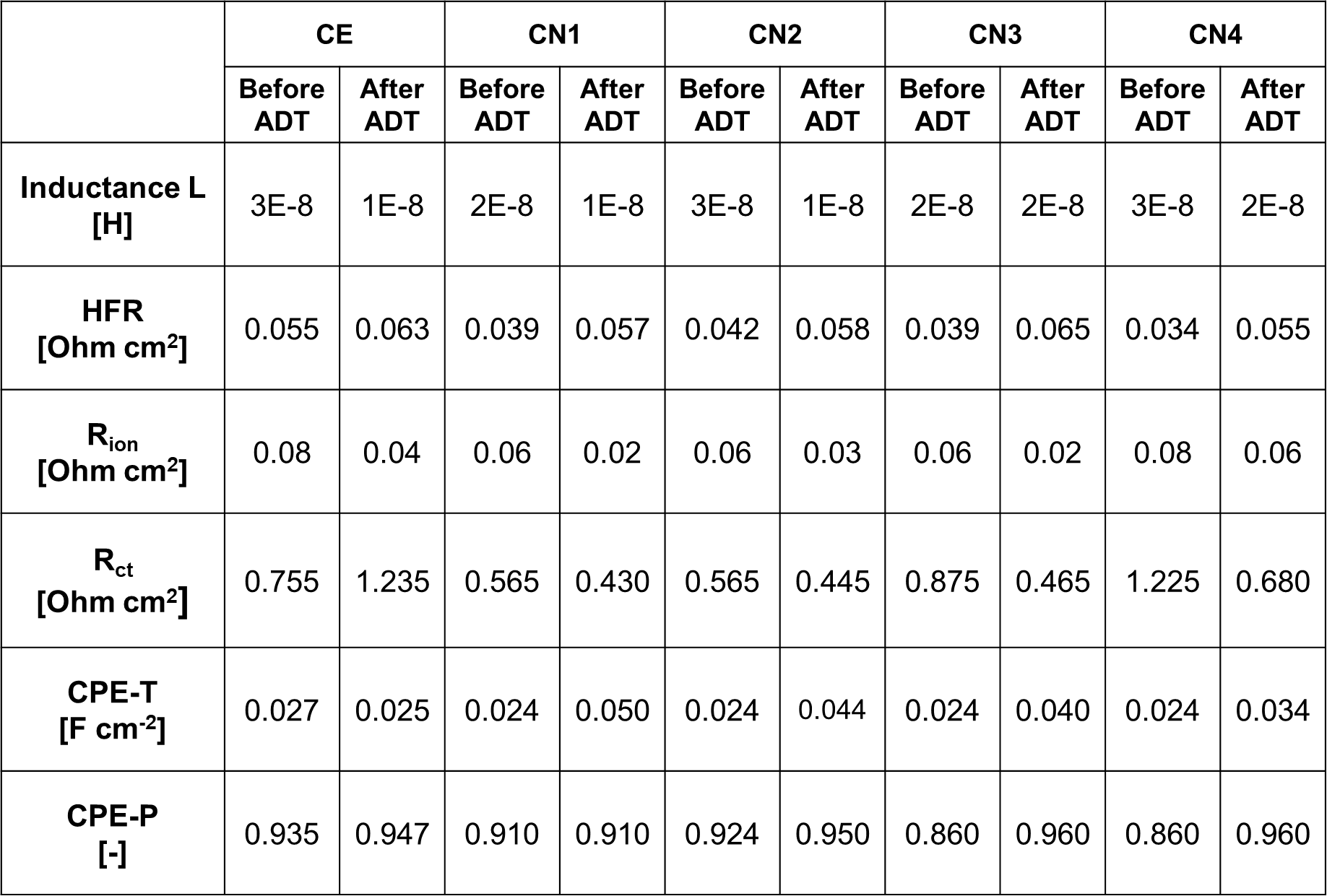


**Supplementary Note 1: Overpotential analysis**

The three main sources of overpotential, namely, (i) ohmic, (ii) mass transfer, and (iii) kinetic overpotential, are generally known to determine the performance of PEMFCs. The contribution of each polarization source was quantitatively evaluated by performing an overpotential analysis for each electrode at a current density of 1.5 A cm^−2^. The overpotential from ohmic loss, *Ƞ*_ohmic_, is calculated directly using the impedance (i.e., the high-frequency resistance measurement from EIS analysis). The overpotential from limited mass transfer, *Ƞ*_mass_, is estimated based on the cell voltage gap between the kinetic-controlled potential for the ideal Tafel slope and catalyst activity (*E*_kinetic_) and the iR-corrected electrochemical potential (*E*_iR-corrected_); *E*_0_ denotes the equilibrium potential at the operating temperature and unit activity of the reactants and products, and *E*_kinetic_ denotes the Tafel slope, which is estimated in the absence of ohmic and mass transfer effects.^19^ The remainder of the overpotential, after subtracting the contribution of *Ƞ*_ohmic_ and *Ƞ*_mass_, is considered to be from the kinetic loss, that is, *Ƞ*_kinetic_. The equations for each expression are given below, and the results are shown in **Fig. 4a**.

*Ƞ*_kinetic_ = *E*_0_ – *E*_kinetic_

*Ƞ*_ohmic_ = *E*_iR-corrected_ – *E*_cell_

*Ƞ*_mass_ = *E*_kinetic_ – *E*_iR-corrected_

**Supplementary Note 2: Normerized cell performance compared to other references**

Figure S13 presents the normalized initial and cell performance after ADT of each electrode relative to its corresponding conventional Pt/C baseline, enabling a direct comparison of initial performance and durability. The degradation rate of the commercial Pt/C electrode in this study (−57.7%) was comparable to those reported under DOE catalyst support AST protocols (−57.0% to −64.5%) ^[31, 64]^, validating the reliability of our comparative analysis. Despite variations in experimental conditions such as gas stoichiometry, backpressure, and ADT protocols among different studies, several representative references were selected for comparison.

Most previous studies have aimed to replace catalyst supports with corrosion-resistant materials such as graphitic carbons (e.g., GC, CNF, and CNT) and metal oxides (e.g., TiO_2_). ^[15, 17, 21, 31, 32, 64]^ While these alternative supports have demonstrated lower degradation rates, they often suffer from limited initial performance, making practical application challenging. In contrast, this study employs a different approach by using commercial Pt/C catalysts combined with CNT and NT composites as structural additives, thereby avoiding the need to develop entirely new support materials. With this new concept, CN2 exhibited significantly lower degradation while maintaining high initial performance compared to other electrodes.

Therefore, this study demonstrate that the structural reinforcement using CNT and NT composites offers a practical and effective strategy to enhance both the durability and performance of PEMFC electrodes, without compromising either aspect or requiring additional synthesis steps to replace the catalyst support.
